# The *Saccharomyces cerevisiae* Spo7 basic tail is required for Nem1–Spo7/Pah1 phosphatase cascade function in lipid synthesis

**DOI:** 10.1016/j.jbc.2023.105587

**Published:** 2023-12-21

**Authors:** Ruta Jog, Gil-Soo Han, George M. Carman

**Affiliations:** Department of Food Science and the Rutgers Center for Lipid Research, New Jersey Institute for Food, Nutrition, and Health, Rutgers University, New Brunswick, New Jersey, USA

**Keywords:** Nem1, Spo7, Pah1, protein phosphatase, phosphatidate phosphatase, diacylglycerol, triacylglycerol, lipid droplet, membrane, phospholipid, yeast, *Saccharomyces cerevisiae*

## Abstract

The *Saccharomyces cerevisiae* Nem1–Spo7 protein phosphatase complex dephosphorylates and thereby activates Pah1 at the nuclear/endoplasmic reticulum membrane. Pah1, a phosphatidate phosphatase catalyzing the dephosphorylation of phosphatidate to produce diacylglycerol, is one of the most highly regulated enzymes in lipid metabolism. The diacylglycerol produced in the lipid phosphatase reaction is utilized for the synthesis of triacylglycerol that is stored in lipid droplets. Disruptions of the Nem1–Spo7/Pah1 phosphatase cascade cause a plethora of physiological defects. Spo7, the regulatory subunit of the Nem1–Spo7 complex, is required for the Nem1 catalytic function and interacts with the acidic tail of Pah1. Spo7 contains three conserved homology regions (CR1–3) that are important for the interaction with Nem1, but its region for the interaction with Pah1 is unknown. Here, by deletion and site-specific mutational analyses of Spo7, we revealed that the C-terminal basic tail (residues 240-259) containing five arginine and two lysine residues is important for the Nem1–Spo7 complex–mediated dephosphorylation of Pah1 and its cellular function (triacylglycerol synthesis, lipid droplet formation, maintenance of nuclear/endoplasmic reticulum membrane morphology, and cell growth at elevated temperatures). The glutaraldehyde cross-linking analysis of synthetic peptides indicated that the Spo7 basic tail interacts with the Pah1 acidic tail. This work advances our understanding of the Spo7 function and the Nem1–Spo7/Pah1 phosphatase cascade in yeast lipid synthesis.

The yeast *Saccharomyces cerevisiae* is an ideal model to elucidate the metabolism, cell biology, and regulation of eukaryotic lipids. The homology of yeast proteins, biosynthetic pathways, and regulatory networks with those of higher eukaryotic organisms, including humans, provides valuable insights into lipid-based diseases (1, 2, 3). Phosphatidate (PA) is a minor membrane phospholipid in *S. cerevisiae* that plays multiple roles in lipid synthesis at the nuclear/endoplasmic reticulum (ER) membrane (4, 5, 6) (Fig. 1*A*). On the one hand, PA serves as the precursor of all major membrane phospholipids *via* the liponucleotide CDP-diacylglycerol (DAG) (1, 6, 7). On the other hand, PA serves as the precursor of the storage lipid triacylglycerol (TAG) *via* DAG (1, 6, 7, 8). The utilization of PA for the synthesis of the diverse lipids is governed by nutritional status, growth phase, and gene mutations (1, 6, 7). For example, PA is primarily utilized for membrane phospholipid synthesis when yeasts are actively growing, but it is mainly utilized for TAG synthesis when the cells progress into stasis (8, 9). In addition to its use for lipid synthesis, PA regulates the expression of UAS_INO_-containing phospholipid synthesis genes *via* the Henry (Opi1 [repressor]/Ino2–Ino4 [activator complex]) regulatory circuit by sequestering Opi1, in concert with Scs2, at the nuclear/ER membrane (1, 6, 7, 10, 11, 12). Indeed, elevated PA content is consistent with an increase in phospholipid synthesis *via* the derepression of phospholipid synthesis gene expression (1, 6, 7).

**Figure 1:**
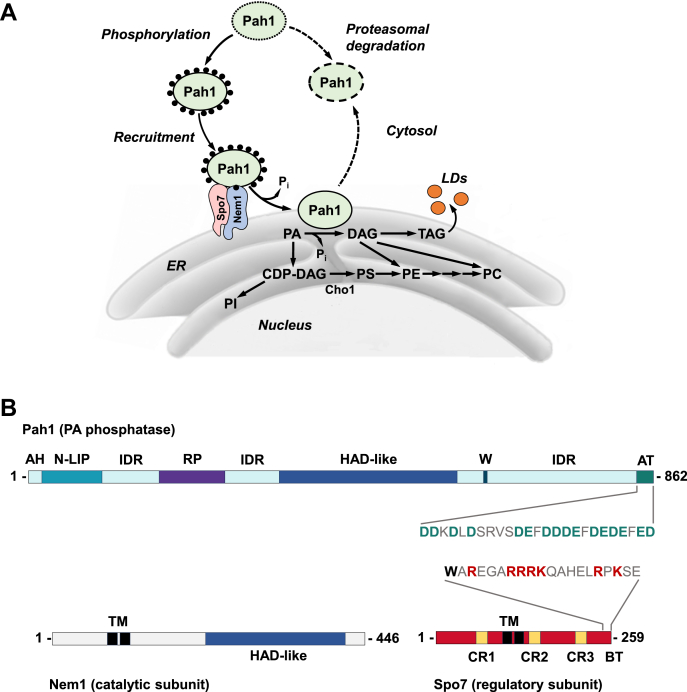
**Model for Pah1 recruitment and dephosphorylation by the Nem1–Spo7 protein phosphatase complex and schematics for Pah1, Nem1, and Spo7 domains/regions.***A*, the recruitment and dephosphorylation of the cytosolic-associated phosphorylated (*black circles*) Pah1 at the nuclear/ER membrane is mediated by its interaction and dephosphorylation by the Nem1–Spo7 protein phosphatase complex. After its association with the membrane, Pah1 binds to and dephosphorylates its substrate PA to form DAG, which is then acylated to TAG that is subsequently stored in cytosolic lipid droplets. PA can also be diverted to the synthesis of phospholipids *via* CDP-DAG when Pah1 activity is reduced. Unphosphorylated/dephosphorylated Pah1 is unstable (*dotted lines*) and undergoes proteasomal degradation. *B*, the linear schematics show domains/regions of Pah1 (*upper*), Nem1 (*lower left*), and Spo7 (*lower right*). The N-terminal amphipathic helix (*AH*) of Pah1 is required for its interaction with the membrane (34), the catalytic core is composed of the conserved N-LIP and haloacid dehalogenase (HAD)–like domains (13, 47, 104), the conserved RP domain that regulates the phosphorylation state of Pah1 (79), a conserved tryptophan (*W*) residue for Pah1 function *in vivo* (105), and the acidic tail (*AT*) (negatively charged residues highlighted in *green*) that is required for interaction with the Nem1–Spo7 complex (35). Nem1 contains transmembrane (*TM*) regions and the haloacid dehalogenase (*HAD*)–like catalytic domain (30). Spo7 contains transmembrane regions, three conserved regions, *CR1–3*, which are involved in its interaction with Nem1 (42, 43), and the basic tail (*BT*) (positively charged residues highlighted in *red*), which are involved in its interaction with Pah1 (this study). CDP-DAG, cytidine diphosphate diacylglycerol; Cho1, phosphatidylserine synthase; ER, endoplasmic reticulum; LD, lipid droplet; PA, phosphatidate; PC, phosphatidylcholine; PE, phosphatidylethanolamine; PI, phosphatidylinositol; PS, phosphatidylserine; TAG, triacylglycerol.

Among the enzymes that control the formation of PA (*e.g.*, lysoPA acyltransferase, DAG kinase, phospholipase D) and its utilization (*e.g.*, PA phosphatase, CDP-DAG synthase) (1, 5, 6), Pah1 PA phosphatase (13) has emerged as a key regulator that controls PA levels and its bifurcation into phospholipids and TAG (6, 7, 14, 15) (Fig. 1*A*). The phosphatase enzyme is regulated by mechanisms that control its gene expression (16, 17) and biochemical function (18, 19, 20, 21). More importantly, Pah1 is subject to the post-translational modifications of phosphorylation and dephosphorylation that control its localization, PA phosphatase activity, and protein stability (7, 15, 22) (Fig. 1*A*). The phosphorylation of Pah1 occurs on multiple serine–threonine residues that are largely located within the intrinsically disordered regions of the protein (Fig. 1*B*). The protein kinases that catalyze Pah1 phosphorylation have been characterized, and they include the cyclin-dependent kinases Pho85 (23) and Cdc28 (24), casein kinases I (25) and II (26), protein kinases A (27) and C (28), and the glycogen synthase kinase Rim11 (29). The kinase-specific target sites are not only generally distinct but also overlapping for different protein kinases (22). In some sites of Pah1, its phosphorylation is hierarchical in nature (*e.g.*, phosphorylation of one site affects the phosphorylation of another site). Phosphorylated Pah1 is stable against its proteasomal degradation but sequestered in the cytosol apart from its substrate present at the nuclear/ER membrane (22) (Fig. 1*A*). The multiple phosphorylation of Pah1 also has an inhibitory effect on its PA phosphatase activity (23, 27). The effects of Pah1 phosphorylation are complicated in that some protein kinases promote its proteasomal degradation or stimulate its PA phosphatase activity. There are also many sites of Pah1 phosphorylation for which the protein kinase involved has yet to be identified (22).

In contrast to Pah1 phosphorylation by multiple protein kinases, its dephosphorylation is catalyzed by a single protein phosphatase that is composed of the Nem1 (catalytic) and Spo7 (regulatory) subunits (30, 31, 32, 33) (Fig. 1, *A* and *B*). The Nem1–Spo7 phosphatase complex has two functions; it recruits Pah1 to the nuclear/ER membrane and dephosphorylates the enzyme (22, 31, 32, 33, 34, 35) (Fig. 1*A*). The dephosphorylation permits Pah1 to associate with the membrane for its enzyme activity on PA (36) (Fig. 1*A*). In addition, the dephosphorylation of Pah1 stimulates its PA phosphatase activity (33). The Nem1–Spo7 complex acting on Pah1 is also regulated post-translationally for its enzyme activity. The phosphatase complex is stimulated by the Pah1 substrate PA (37) but inhibited by the ER-associated protein Ice2 (38). Like Pah1, the Nem1 and Spo7 subunits are both subject to phosphorylation by protein kinases A (39) and C (40). The phosphorylation of the Nem1–Spo7 complex by these protein kinases has opposing effects on its enzyme activity and cellular function such as TAG synthesis; protein kinase A inhibits the protein phosphatase activity (39), whereas protein kinase C stimulates the enzyme activity (40).

Spo7 plays an important regulatory role in the Nem1–Spo7/Pah1 phosphatase cascade. By forming a complex with Nem1, which is mediated by its three conserved regions (CR1, CR2, and CR3) (Fig. 1*B*), Spo7 promotes the function of Nem1 to dephosphorylate Pah1 (30, 38, 41, 42). Spo7 also interacts with the acidic tail of Pah1 (Fig. 2*B*) for its recruitment at the nuclear/ER membrane (35, 41). However, the region of Spo7 that is responsible for interaction with the acidic tail has been unknown. In the current study, we identified that basic amino acids in the C-terminal region of Spo7 are involved in the interaction with the acidic tail of Pah1. This finding advances the understanding of the Spo7 function in the Nem1–Spo7 phosphatase complex, which activates Pah1 PA phosphatase.

**Figure 2:**
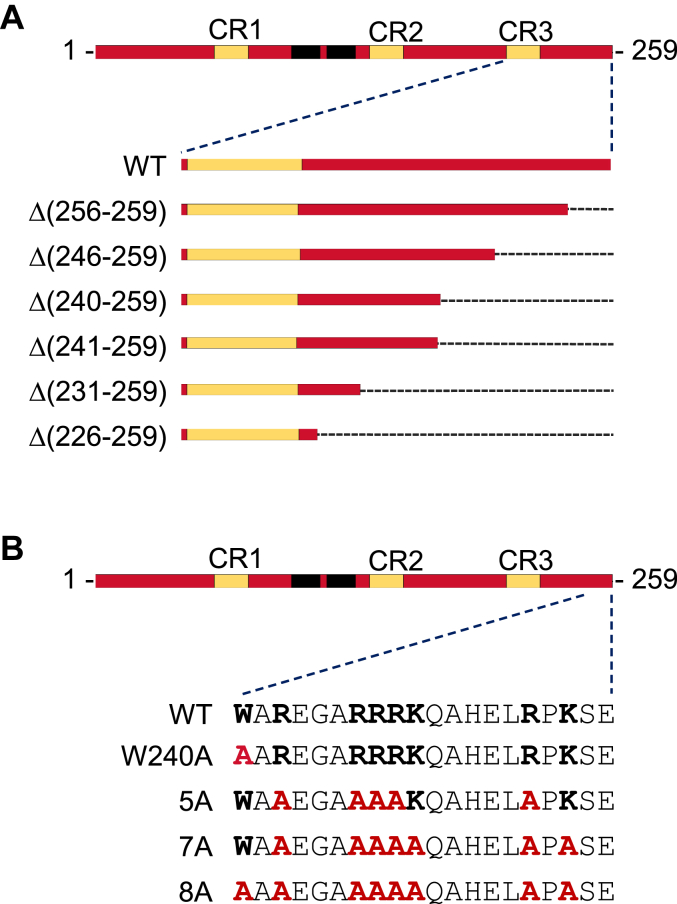
**Schematic representation of C-terminal region mutant forms of Spo7.** The diagrams illustrate the Spo7 C-terminal deletion (*A*) and site-specific (*B*) mutant constructs used in this work.

## Results

### Spo7 basic tail is required for the Nem1–Spo7/Pah1 phosphatase cascade function

We undertook a systematic approach to identify the C-terminal region of Spo7 for its functional significance. For this analysis, a series of deletions were generated from the C-terminal end of *SPO7* (Table 1, Fig. 2*A*), and the mutant alleles were expressed under the control of the native promoter on a low-copy plasmid in a *spo7*Δ mutant strain (Table 1). The loss of Spo7 function, and hence the Nem1–Spo7/Pah1 phosphatase cascade, exhibits the same defects caused by the lack of Pah1 function, which include a decrease in TAG levels, reduced lipid droplet numbers, a growth defect at elevated temperature, and a defect in nuclear morphology (42, 43). Accordingly, the Spo7 deletion mutants were analyzed for their *in vivo* function to complement the *spo7*Δ phenotypes.

**Table 1:** Plasmids and strains used in this study

Plasmid or strain	Relevant characteristics	Source or reference
Plasmid		
YCplac111	Single-copy number *E. coli*/yeast shuttle vector with *LEU2*	(103)
Derivative		
YCplac111-*GAL1/10*-*NEM1*-PtA	*NEM1*-PtA under control of *GAL1/10* promoter inserted into YCplac111	(31)
pRS414	Single-copy number *E. coli*/yeast shuttle vector with *TRP1*	(87)
Derivative		
pGH450	*SEC63-GFP* fusion inserted into pRS414	This study
pRS415	Single-copy number *E. coli*/yeast shuttle vector with *LEU2*	(87)
Derivatives		
pGH443	*SPO7* inserted into pRS415	(39)
pGH443-Δ(256-259)	*SPO7* lacking 256-259	This study
pGH443-Δ(246-259)	*SPO7* lacking 246-259	This study
pGH443-Δ(241-259)	*SPO7* lacking residues 241-259	This study
pGH443-Δ(240-259)	*SPO7* lacking residues 240-259	This study
pGH443-Δ(231-259)	*SPO7* lacking residues 231-259	This study
pGH443-Δ(226-259)	*SPO7* lacking residues 226-259	This study
pGH443-W240A	*SPO7* with the W240A mutation	This study
pGH443-5A	*SPO7* with the R242A/R246A/R247A/R248A/R255A mutations	This study
pGH443-7A	*SPO7* with the R242A/R246A/R247A/R248A/R255A/K249A/K257A mutations	This study
pGH443-8A	*SPO7* with the W240A/R242A/R246A/R247A/R248A/R255A/K249A/K257A mutations	This study
pRS314	Single-copy number *E. coli*/yeast shuttle vector with *TRP1*	(87)
Derivatives		
pRS314-*GAL1/10-SPO7*	*SPO7* under the control of *GAL1/10* promoter inserted in pRS314	(39)
pRS314-*GAL1/10-SPO7*-7A	*SPO7* with the R242A/R246A/R247A/R248A/R255A/K249A/K257A mutations	This study
pRS314-*GAL1/10-SPO7*-8A	*SPO7* with the W240A/R242A/R246A/R247A/R248A/R255A/K249A/K257A mutations	This study
pGH452	*PAH1*-PtA under the control of *GAL1* promoter derived from high-copy number *E. coli*/yeast shuttle vector pYES2	(88)
Strain		
*E. coli*		
DH5α	F^-^ φ80d*lacZ*ΔΜ15Δ (*lacZYA*-*argF*)U169 *deoR rec*A1 *end*A1 *hsd*R17(*r*_k_^-^*m*_*k*_^+^) *pho*A *sup*E44 λ^−^*thi-*1 *gyr*A96 *rel*A1	(83)
*S*. *cerevisiae*		
RS453	*MAT***a***ade2-1 his3-11,15 leu2-3112 trp1-1 ura3-52*	(89)
Derivatives		
GHY67	*spo7*Δ*::URA3*	(39)
SS1010	*nem1::HIS3 spo7::HIS3*	(30)
GHY85	*nem1::HIS3 spo7::HIS3 pah1*Δ*::natMX4*	(43)
SS1132	*pah1*Δ*::TRP1 nem1*Δ*::HIS3*	(24)

**Figure 3:**
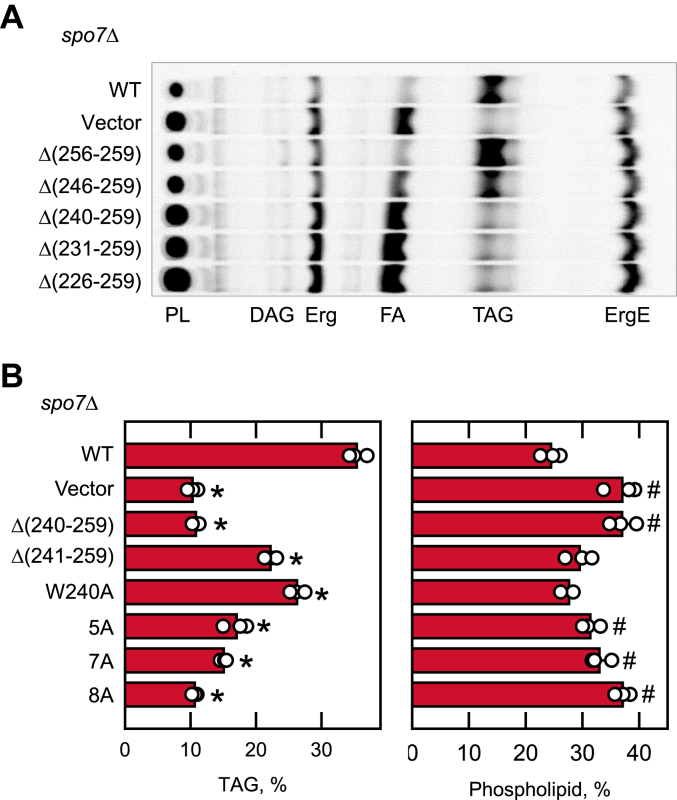
**Lipid composition of *spo7*Δ cells expressing C-terminal region mutant forms of Spo7.** The *spo7*Δ mutant (GHY67) was transformed with pGH443 or its derivatives for the expression of the WT and the C-terminal region deletion and site-specific mutant forms of Spo7. The transformants were grown at 30 ^°^C to the stationary phase in SC-Leu medium containing [2-^14^C]acetate (1 μCi/ml). Lipids were extracted from the radiolabeled cells, separated by one-dimensional TLC, and subjected to phosphorimaging. *A*, the chromatogram is representative of biological duplicates. *B*, chromatograms from biological triplicates of a separate experiment were subjected to ImageQuant analysis; the levels of TAG and phospholipids were normalized to total chloroform-soluble lipids. The data are means ± SD (*error bars*); the individual data points are also shown in the *panel.* ∗*p* < 0.05 *versus* TAG of WT cells. #*p* < 0.05 *versus* phospholipid of WT cells. Erg, ergosterol; ErgE, ergosterol ester; FA, fatty acid; PL, phospholipid; TAG, triacylglycerol.

For lipid analysis, *spo7*Δ cells expressing the *SPO7* allele were grown with [2-^14^C]acetate to the stationary phase when TAG levels are the highest (13, 16, 44). The TLC separation of the radiolabeled lipids showed that the TAG level was substantially lower in *spo7*Δ cells when compared with those expressing WT *SPO7* (Fig. 3*A*). As anticipated, the mutant cells containing decreased TAG levels showed a reciprocal increase in phospholipid levels (42, 43, 44). The expression of the *spo7*-Δ(256-259) allele restored the TAG level of *spo7*Δ cells comparable to that of the mutant cells expressing WT *SPO7* (Fig. 3*A*), whereas the *spo7*-Δ(246-259) allele partially restored the TAG level. However, the *spo7* alleles with larger deletions (*e.g.*, Δ(240-259), Δ(231-259), and Δ(226-259)) did not increase the TAG level. These results indicate that the C-terminal deletion of 20 amino acid residues (*i.e.*, Δ(240-259)) is sufficient to abolish the Spo7 function.

The C-terminal region (residues 240-259) of Spo7 features basic amino acids (five arginine and two lysine residues), which raises a possibility that the basic tail forms ionic interactions with the Pah1 acidic tail. The C-terminal region also contains a tryptophan residue, which plays a role in anchoring membrane proteins into the lipid bilayer (45, 46). To determine if the basic and aromatic amino acids are important for Spo7 function, we mutated them to nonpolar aliphatic alanine residues (Table 1, Fig. 2*B*). Compared with *spo7*Δ cells expressing WT *SPO7*, the mutant cells expressing the 5A and 7A alleles contained 2- and 2.4-fold, respectively, lower levels of TAG (Fig. 3*B*). This result showed that the mutations of all basic amino acids greatly reduce the function of *SPO7* in TAG synthesis. In contrast, *spo7*Δ cells expressing the W240A allele contained a 1.3-fold lower level of TAG, indicating that the aromatic amino acid mutation moderately reduced the *SPO7* function. However, the expression of the 8A allele did not increase the TAG level of *spo7*Δ cells, indicating that the combined mutations have an additive effect and completely abolish Spo7 function.

**Figure 4:**
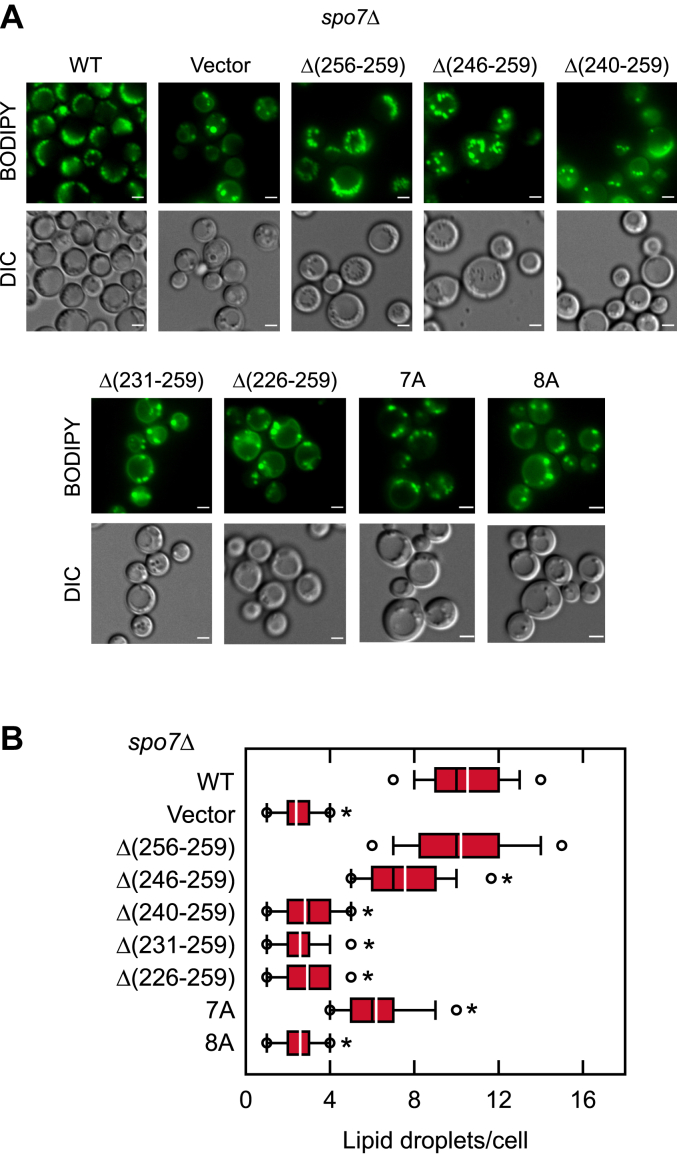
**Lipid droplet formation of *spo7*Δ cells expressing C-terminal region mutant forms of Spo7.** The *spo7*Δ mutant (GHY67) was transformed with pGH443 or its derivatives for the expression of the WT and the C-terminal region truncation and site-specific mutant forms of Spo7. The cells were grown at 30 ^°^C in SC-Leu medium to the stationary phase and then stained with BODIPY 493/503. The stained lipid droplets were visualized by fluorescence microscopy, and the number of lipid droplets was counted from ≥200 cells (≥4 fields of view). *A*, the images shown are representative of multiple fields of view. Scale bar represents 1 μm. In some fields of view, the mutant cells look larger than the WT. The reason for this is unclear. *B*, the data are presented by the box plot. The *black* and *white lines* are the median and mean values, respectively, and the *white circles* are the outlier data points of the 5th and 95th percentiles. ∗*p* < 0.05 *versus* WT. DIC, differential interference contrast.

The effects of the Spo7 basic tail mutations on cytoplasmic lipid droplets was examined by staining cells with the fluorescent lipophilic dye BODIPY 493/503 (Fig. 4). The *spo7*Δ cells expressing the vector control showed an approximately three fold reduction in cellular lipid droplets when compared with the *spo7*Δ cells expressing the WT *SPO7*. In line with their impact on cellular TAG content, the *spo7*Δ cells expressing the C-terminal deletion and site-specific mutations beginning at residue 240 showed reductions in lipid droplet numbers (Fig. 4). For example, the cells expressing the 8A mutations had a three fold reduction in lipid droplet number when compared with the WT control.

**Figure 5:**
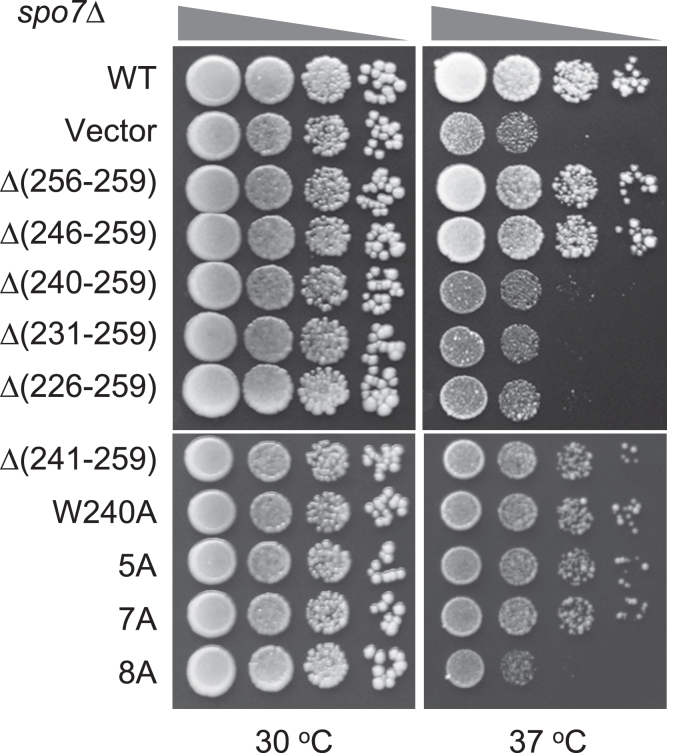
**Temperature sensitivity of *spo7*Δ cells expressing C-terminal region mutant forms of Spo7.** The *spo7*Δ mutant (GHY67) was transformed with pGH443 or its derivatives for the expression of the WT and the C-terminal region truncation and site-specific mutant forms of Spo7. The transformants were grown to saturation at 30 ^°^C in SC-Leu medium. The cultures were adjusted to absorbance of 0.7 at 600 nm, serially diluted (10-fold) in SC-Leu medium, and spotted (2.5 μl) onto YPD plates. Colony growth at 30 and 37 ^°^C was scored after 3 days of incubation. The data are representative of biological triplicates. YPD, yeast extract, peptone, and dextrose.

Loss of Spo7 function (and Nem1–Spo7/Pah1 phosphatase cascade) in lipid synthesis is characterized by the inability of mutant cells to grow at the restrictive temperature of 37 ^°^C (6). As described previously (43), WT Spo7 complemented the temperature-sensitive phenotype imparted by the *spo7*Δ mutation (Fig. 5). Consistent with the previous results, the WT and Δ(256-259) and Δ(246-259) mutant forms of Spo7 complemented the temperature-sensitive phenotype of the *spo7*Δ mutant, whereas the mutants with the deletions from residue 240 and beyond failed to complement this phenotype. Of the point mutations, 8A had the greatest effect by failing to complement the temperature-sensitive phenotype characteristic of the *spo7*Δ mutant (Fig. 5). In subsequent experiments, we limited our analysis to the 7A and 8A mutations.

**Figure 6:**
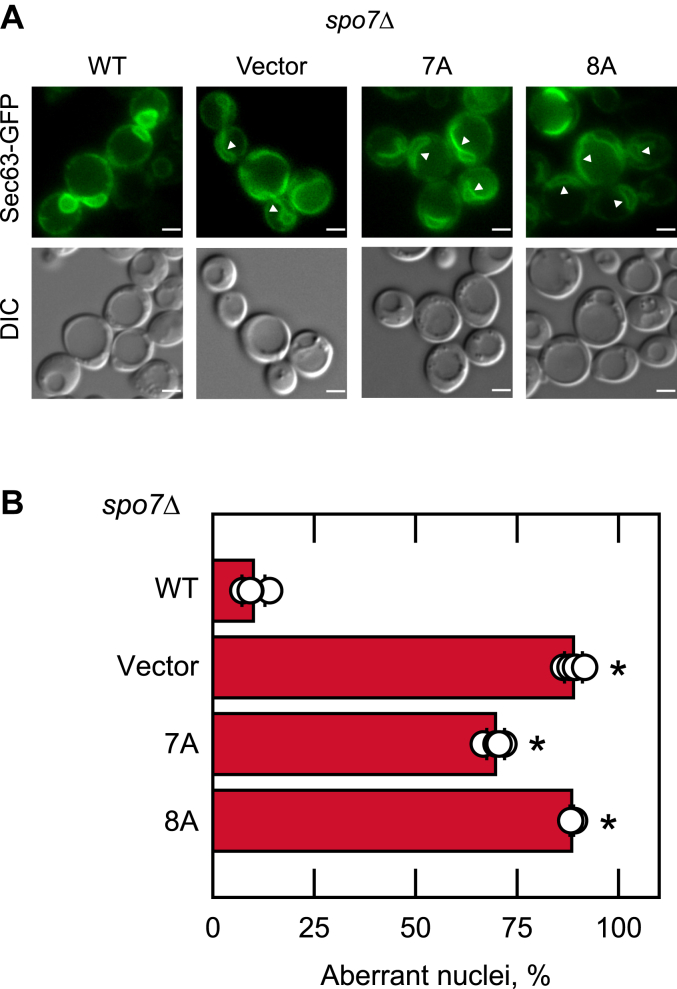
**Nuclear morphology of *spo7*Δ cells expressing C-terminal region mutant forms of Spo7.***A*, the *spo7*Δ mutant (GHY67) was transformed with pGH450 (*SEC63-GFP* nuclear membrane marker) and pGH443 or its derivatives for the expression of the WT and the C-terminal 7A and 8A mutant forms of Spo7. The cells were grown at 30 ^°^C in SC-Leu-Trp medium to the exponential phase of growth. The images shown are representative of multiple fields of view. The *white arrow heads* indicate aberrant nuclei. Scale bar represents 1 μm. *B*, the percentage of aberrant nuclei are presented from ≥200 cells (≥4 fields of view). The data are averages ± SD (*error bars*). ∗*p* < 0.05 *versus* aberrant nuclei of WT cells. DIC, differential interference contrast.

*spo7*Δ cells defective in the Nem1–Spo7/Pah1 phosphatase cascade exhibit the aberrant expansion of the nuclear/ER membrane with irregularly shaped nuclei (30, 31, 42). This phenotype is ascribed to the increase in phospholipid synthesis that occurs in response to the defect in the Nem1–Spo7/Pah1-mediated synthesis of TAG (31, 47). WT Spo7 complements this phenotype as most cells have round nuclei (42) (Fig. 6). The Spo7 C-terminal 7A and 8A mutations failed to complement this phenotype; 70 and 88%, respectively, of the *spo7*Δ cells expressing these mutations exhibited abnormally shaped nuclei (Fig. 6).

### Spo7 basic tail mutations cause the derepression of *CHO1*-encoded phosphatidylserine synthase

**Figure 7:**
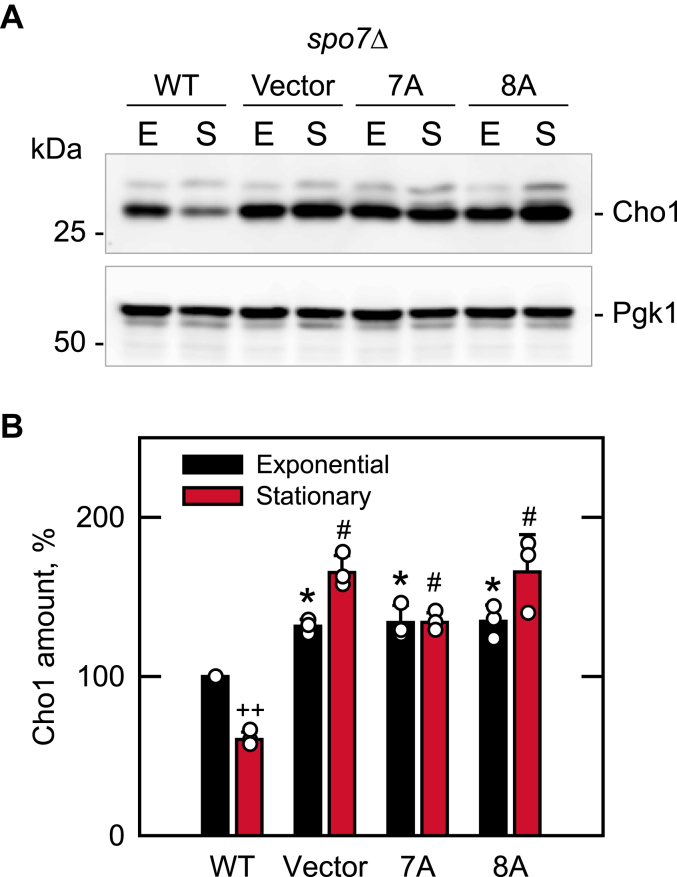
**Cho1 expression of *spo7*Δ cells expressing C-terminal region mutant forms of Spo7.***A*, the *spo7*Δ mutant (GHY67) was transformed with pGH443 or its derivatives for the expression of the WT and C-terminal 7A and 8A mutant forms of Spo7. The transformants were grown to the exponential (*E*) and stationary (*S*) phases in SC-Leu medium at 30 ^°^C. Cell extracts were prepared, the proteins were resolved by SDS-PAGE, and transferred to a polyvinylidene difluoride membrane, which was cut for separately probing for Cho1 and Pgk1 (loading control), with rabbit anti-Cho1 and mouse anti-Pgk1 antibodies, respectively. The positions of Cho1, Pgk1, and molecular mass standards are indicated. The minor band above the Cho1 band is a phosphorylated form of the protein (81). The immunoblots are representative of biological triplicates. *B*, the relative amount of Cho1 was determined by ImageQuant analysis of three independent experiments ± SD (*error bars*). The individual data points are also shown. ∗*p* < 0.05 *versus* WT from exponential phase cells. ^#^*p* < 0.05 *versus* WT from stationary phase cells. ^++^*p* < 0.05 stationary *versus* exponential phase of WT cells.

The increase in phospholipid synthesis and the aberrant expansion of the nuclear/ER membrane imparted by mutations in the Nem1–Spo7/Pah1 phosphatase cascade is rooted, at least in part, in the PA-mediated derepression of UAS_INO_-containing phospholipid synthesis genes *via* the Henry (Opi1/Ino2–Ino4) regulatory circuit (1, 6, 7, 11, 31, 32, 48). One of the genes that is particularly affected by the *pah1*Δ mutation, which elevates the level of PA and disorders the regulation of phospholipid synthesis (13, 31), is *CHO1* (49). *CHO1* encodes phosphatidylserine synthase (50), the enzyme that catalyzes the first step in the synthesis of the major membrane phospholipid phosphatidylcholine *via* the CDP-DAG pathway (1, 6, 7, 48) (Fig. 1*A*). During cell growth, the expression of *CHO1* is derepressed in the exponential phase when phospholipid synthesis predominates over TAG synthesis and repressed in the stationary phase when TAG synthesis predominates over phospholipid synthesis (9, 49, 51, 52, 53, 54). As expected, the level of Cho1 from the stationary phase *spo7*Δ cells expressing WT Spo7 was 1.7-fold lower when compared with the same cells in the exponential phase (Fig. 7). Owing that Spo7 regulates Pah1 function *via* the Nem1–Spo7 phosphatase complex (Fig. 1), we questioned whether the *spo7*Δ mutation affects the growth phase–mediated regulation of Cho1 expression (Fig. 7). The *spo7*Δ mutation disrupted the normal pattern of Cho1 expression; the Cho1 level was not reduced in the stationary phase. In fact, the Cho1 levels in the *spo7*Δ mutant cells in the exponential and stationary phases were elevated by 1.3- and 2.7-fold, respectively, when compared with the levels in *spo7*Δ cells expressing WT Spo7 (Fig. 7). The Spo7 C-terminal 7A and 8A mutations failed to correct this phenotype; the levels of Cho1 were elevated in both phases of growth when compared with the levels in *spo7*Δ cells expressing WT Spo7 (Fig. 7).

### Spo7 basic tail is required for Nem1–Spo7 protein phosphatase activity on Pah1

**Figure 8:**
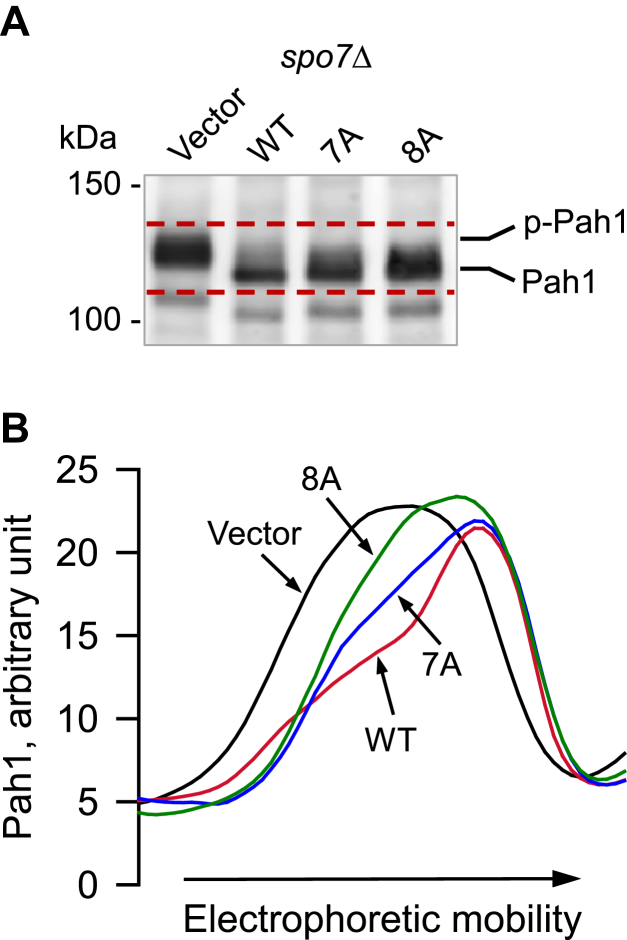
**Effect of Spo7 C-terminal region mutations on the Nem1–Spo7-mediated dephosphorylation of Pah1.** Phosphorylated Pah1 (2.5 ng) was incubated with membranes (20 μg) prepared from *nem1*Δ *spo7*Δ *pah1*Δ (GHY85) cells coexpressing plasmids YCplac111-*GAL1/10-NEM1*-PtA and pRS314-*GAL1/10-SPO7* (WT or C-terminal 7A and 8A mutant forms) under the assay conditions for Nem1–Spo7 phosphatase activity (33). Following the incubation, the reaction mixtures were resolved by SDS-PAGE (6% polyacrylamide gel), transferred to polyvinylidene difluoride membrane, and probed with anti-Pah1 antibody. *A*, the positions of Pah1 in the phosphorylated (*p-Pah1*) and dephosphorylated (*Pah1*) states and molecular mass standards are indicated. The *dashed red line* is a guide to show the range in the electrophoretic mobility of Pah1 in cells expressing the WT and mutant forms of Spo7. *B*, the signal intensities of Pah1 along its migration in the region between the *dashed red lines* in *panel A* were measured using the line graph function of ImageQuant software. The densitogram of Pah1 mobility after incubation with membranes expressing the WT (*red line*) and 7A (*blue line*) and 8A (*green line*) mutant forms of Spo7 and vector (*black line*). The data shown are representative of triplicate experiments.

Spo7 is required for the Nem1 catalytic activity to dephosphorylate Pah1 (30, 42, 43). Accordingly, we questioned whether this function of Spo7 is affected by its C-terminal region mutations. To determine the mutational effect, the hyperphosphorylated preparation of Pah1 was incubated with the membranes isolated from *pah1*Δ *nem1*Δ *spo7*Δ triple mutant cells overexpressing both Nem1 and Spo7 (WT or C-terminal region mutant forms) (33). The dephosphorylation of Pah1 by the Nem1–Spo7 phosphatase was assessed by an increase in its electrophoretic mobility upon SDS-PAGE (23, 24, 42, 55). As described previously (42, 43), Pah1 incubated with the membranes containing WT Spo7 showed faster electrophoretic mobility when compared with the protein incubated with the Spo7-free membranes (vector control) (Fig. 8). In addition, dephosphorylated Pah1 was less abundant than the unphosphorylated form (vector control) (Fig. 8); the dephosphorylation of Pah1 renders the protein unstable and subject to proteolytic degradation *via* the 20S proteasome (56, 57). When incubated with the membranes containing the C-terminal region mutant forms of Spo7, Pah1 had a slower electrophoretic mobility and was qualitatively more abundant (Fig. 8). The 8A mutant had little effect on the electrophoretic mobility of Pah1, whereas the 7A mutant showed a partial effect. These electrophoretic patterns supported the conclusion that the C-terminal basic residues of Spo7 are important for the Nem1–Spo7 protein phosphatase activity.

### Complex formation of Spo7 with Nem1 does not require its basic tail

**Figure 9:**
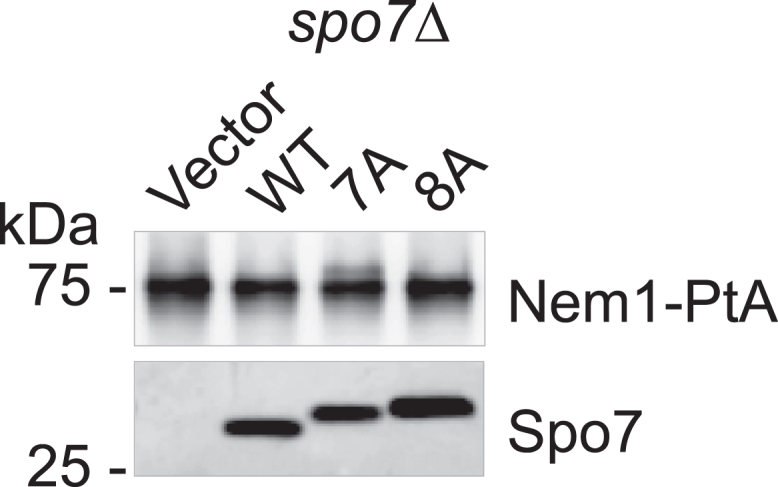
**Complex formation of Nem1 with C-terminal region mutant forms of Spo7.** Protein A-tagged Nem1 was isolated from the cell extracts of *nem1*Δ *spo7*Δ *pah1*Δ (GHY85) cells coexpressing plasmids YCplac111-*GAL1/10*-*NEM1*-PtA and pRS314-*GAL1/10-SPO7* (WT or C-terminal 7A and 8A mutant forms) using IgG-Sepharose resin (30, 33). The affinity-isolated Nem1 preparations were resolved by SDS-PAGE (12.5% polyacrylamide gel) and transferred to a polyvinylidene difluoride membrane. Sample loading was normalized to similar amounts of Nem1 as determined by a separate immunoblot analysis with anti-protein A antibody. The membrane was split into *upper* and *lower* portions and probed with rabbit anti-protein A and rabbit anti-Spo7 antibodies, respectively. The formation of the Nem1–Spo7 complex was scored by the presence of Spo7 in the affinity-isolated Nem1 preparation (33). The positions of Nem1, Spo7, and molecular mass standards are indicated. The C-terminal mutant forms of Spo7 had a slower electrophoretic mobility when compared with the WT form. The data shown are representative of four replicate experiments.

We questioned whether the C-terminal region mutations of Spo7 affect its complex formation with Nem1. For this analysis, the WT and C-terminal region mutant forms of Spo7 were coexpressed with protein A-tagged Nem1 in *spo7*Δ *nem1*Δ *pah1*Δ triple mutant cells. The protein A-tagged Nem1 was isolated from cell extracts with IgG-Sepharose resin (30, 33). The affinity-isolated Nem1 was resolved by SDS-PAGE and transferred to a polyvinylidene difluoride membrane, which was then probed with anti-protein A and anti-Spo7 antibodies. The formation of the Nem1–Spo7 complex was identified by the presence of Spo7 in the isolated Nem1 preparation (33). The immunoblot analysis showed that the WT and C-terminal region mutant forms of Spo7 were associated with the protein A-tagged Nem1 (Fig. 9). This result indicated that the C-terminal residues 240 to 259 of Spo7 do not affect its interaction with Nem1. We observed that the electrophoretic mobility of the C-terminal mutant forms of Spo7 was slower when compared with that of WT Spo7. The reason for this difference is unclear.

### Spo7 basic tail interacts with Pah1 acidic tail

**Figure 10:**
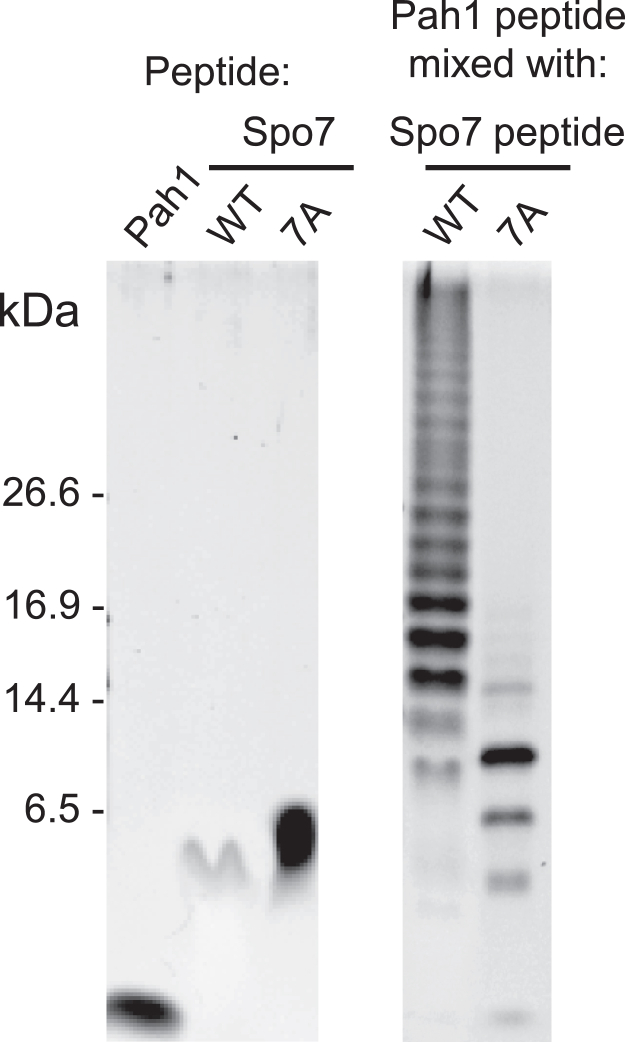
**Crosslinking of Spo7 C-terminal peptide with Pah1 C-terminal peptide.** Spo7 WT (GGGGWAREGARRRKQAHELRPKSE) or 7A mutant (GGGGWAAEGAAAAAQAHELAPASE) C-terminal peptide (20 μg) was mixed with Pah1 (GGGGDDKDLDSRVSDEFDDDEFDEDEFED) C-terminal peptide (20 μg) in the presence of 5 mM glutaraldehyde for 1 h at room temperature. The peptide mixtures were resolved on a tricine–SDS-PAGE (16% polyacrylamide gel), followed by SYPRO ruby staining and fluorescence imaging. The positions of the molecular mass standards are indicated. The Pah1 and Spo7 (WT and 7A mutant) peptides incubated with glutaraldehyde but not mixed with other peptides are shown in the panel on the *left*. For an unknown reason, the WT Spo7 peptide by itself did not stain as well as the other peptides.

Karanasios *et al.* (35) originally showed that the interaction of Pah1 with the Nem1–Spo7 complex is dependent on its C-terminal acidic residues. Subsequent work by Dubots *et al.* (41) indicated that the interaction of Pah1 with Nem1 is dependent on Spo7, whereas the interaction of Pah1 with Spo7 does not require Nem1. We addressed the hypothesis that the Spo7 basic tail (residues 240-259) is a region interacting with the Pah1 acidic tail (residues 838-862). Because this interaction is considered to be noncovalent and transient (58), we performed a glutaraldehyde cross-linking experiment to capture the transient interaction using the synthetic peptides of Spo7 and Pah1 (59) (Fig. 10). Glycine residues were added to the N-terminal Trp-240 of the Spo7 peptide to obviate its potential oxidation that could result in structural changes. Since glycine residues are not expected to increase the hydrophobicity of the peptide or alter the charge, they were also added to the N terminus of the Pah1 peptide. The Spo7 and Pah1 peptides were mixed at equimolar concentrations in the presence of 5 mM glutaraldehyde. After 1 h incubation at room temperature, the peptide mixture was resolved on a tricine–SDS-PAGE. In this analysis, Spo7 and Pah1 peptides were crosslinked to form higher molecular weight oligomers (Fig. 10). However, the oligomer formation was largely prevented when the Pah1 peptide was mixed with the 7A mutant form of the Spo7 peptide. As was expected, the individual peptides of Pah1 and Spo7 were not crosslinked to form oligomers. These results indicated that the C-terminal basic residues of Spo7 interact with the C-terminal acidic residues of Pah1.

## Discussion

In the Nem1–Spo7 complex, Spo7 is the regulatory subunit required for the function of the Nem1 catalytic subunit (30), which together, recruit, dephosphorylate, and stimulate Pah1 function at the nuclear/ER membrane (22, 31, 32, 33, 34, 35) (Fig. 1*A*). In a sense, Spo7 is the master regulator of the Nem1–Spo7/Pah1 phosphatase cascade in that it recruits Pah1 to the complex (35, 41) and stabilizes Nem1 for its catalytic function (30, 38, 41, 42). Although not directly addressed here, Spo7 may also function to position Pah1 for its dephosphorylation by Nem1 and/or provide specificity for Pah1. Given these critical functions, it is not surprising that the *spo7*Δ mutation elicits phenotypes (*e.g.*, reduction in TAG content and lipid droplet formation, aberrant nuclear/ER membrane expansion, vacuole fragmentation, inability to grow on nonfermentable carbon source, and temperature sensitivity) typical of those imparted by the *pah1*Δ mutation (30, 42, 43). These phenotypes result from a disturbance in the PA–DAG balance controlled by the PA phosphatase activity of Pah1 (13, 47).

The structure of Spo7 has not been solved, but its presumed transmembrane domains consist of two hydrophobic stretches located close together (30). As a result, it is plausible that these stretches insert into the membrane resembling a hairpin structure with a short luminal loop (30). This positioning would result in both the N- and C-terminal regions of Spo7 to be oriented toward the cytoplasm (30). Because of their polar nature, C-terminal regions are typically exposed to solvents, making them accessible for protein binding (60). The C-terminal tail of Spo7 is rich in positively charged amino acids (*e.g.*, arginine and lysine residues); we considered that these residues promote the formation of ionic interactions with the acidic tail of Pah1 that is enriched with aspartate and glutamate residues (Fig. 1*B*). The ionic interactions would provide a fast on-rate for Pah1 binding to Nem1–Spo7 (35) so that Pah1 can dissociate from the complex and hop onto the membrane to dephosphorylate its substrate PA to form DAG (36). Previous work has shown the loss of Nem1–Spo7 interaction with Pah1 when its acidic tail is removed (35). To examine the hypothesis, we performed a deletion analysis and found that residues 240 to 259 are critical for Spo7 function in lipid synthesis. This stretch of Spo7 containing seven positively charged amino acids were mutated to alanine residues, which are less likely to cause a severe disruption of the tertiary structure of Spo7. Generally, charged amino acids that are located on the surface of a protein would have a propensity to form hydrogen bonds and ion pairs and consequently may be involved in recognizing interacting proteins (61). Our analysis demonstrated a partial loss of Spo7 function with the 7A mutations. When these mutations were combined with the W240A mutation (*e.g.*, 8A), there was a complete loss of Spo7 function. A possible explanation is that within a membrane protein, tryptophan is typically found at the lipid–water interface, serving as an anchor with a preference for the hydrophilic region over the hydrophobic core (45, 46, 62), and mutating the tryptophan might result in a loss of this membrane anchor and affect the correct positioning of Spo7 C-terminal region with respect to the membrane surface. To directly examine the interaction of the Spo7 basic tail with the Pah1 acidic tail, we employed an established (59) glutaraldehyde cross-linking experiment with synthetic peptides. The results of this experiment revealed an interaction between the Spo7 and Pah1 peptides as shown by formation of oligomeric structures (59). The formation of these oligomers was largely prevented by substituting the WT Spo7 peptide with a Spo7 mutant peptide containing a sequence equivalent to the 7A mutations in full-length Spo7.

The counterpart Nem1–Spo7/Pah1 phosphatase cascade in mammalian cells is called CTDNEP1–NEP1-R1/lipin 1 (63, 64, 65, 66, 67). Lipin 1 is a PA phosphatase (68) whose state of phosphorylation governs its subcellular localization (69, 70, 71, 72, 73), and when expressed in human cells, the CTDNEP1–NEP1-R1 dephosphorylates lipin 1 (67). The critical roles that the phosphatase cascade plays in humans and mice are typified by assorted abnormalities (*e.g.*, lipodystrophy, insulin resistance, peripheral neuropathy, rhabdomyolysis) that result from loss of lipin 1 PA phosphatase activity (63, 74, 75, 76, 77, 78). Yeast Nem1 and its mammalian counterpart CTDNEP1 lack the ability to dephosphorylate Pah1 and lipin 1, respectively, implying that their interaction with their respective specific binding partners, Spo7 and NEP1-R1, is necessary for their catalytic activity (31, 65). Parenthetically, CTDNEP1 is able, however, to dephosphorylate lipin 1 phosphopeptide substrates (66). Spo7 interacts with Nem1 to form a complex *via* its three CRs (42, 43) (Fig. 1*B*). The hydrophobicity imparted by the Leu-54, Leu-55, and Ile-56 (CR1) (42) and Leu-217 and Leu-219 (CR3), and the combination of uncharged hydrophilic residues Ser-141, Thr-142, and Asn-143 (CR2), are all important for the interaction with Nem1 (42, 43). Mutations in any one of the CRs are sufficient to disrupt Nem1–Spo7 complex formation and elicit the deleterious phenotypes that include the defect in TAG synthesis (42, 43). To our knowledge, the importance of CR1–3 in NEP1-R1 for its interaction with CTDNEP1 have not been addressed. It is known that the conserved C-terminal region of Nem1 is involved in complex formation with Spo7 (35), but the specific residues involved have yet to be determined. Work is currently in progress to address this question. Likewise, the residues within CTDNEP1 that are responsible for interaction with NEP1-R1 have not been addressed.

**Figure 11:**
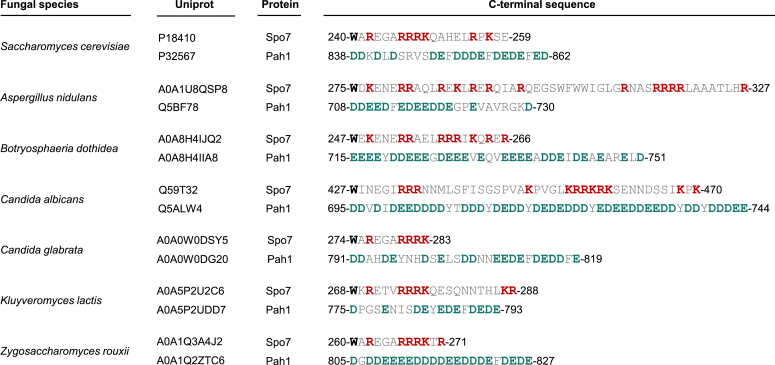
**Fungal sequences of Spo7 basic and Pah1 acidic tails.** The C-terminal Spo7 basic and Pah1 acidic sequences are shown for fungal homologs of Spo7 and Pah1 with the UniProt accession identifications. Positively charged residues in Spo7 homologs, lysine (*K*) and arginine (*R*), are highlighted in *red*. Negatively charged residues in Pah1 homologs, aspartate (*D*) and glutamate (*E*), are highlighted in *green*. The tryptophan (*W*) residue in Spo7 homologs is highlighted in *black*. Residue numbers are indicated at the start and end of each sequence.

The site-specific mutations (*e.g.*, 7A and 8A) of the Spo7 basic tail, which imparted common *spo7*Δ/*pah1*Δ phenotypes, did not disrupt the Nem1–Spo7 complex formation. Thus, the deleterious effects of the basic tail mutations were due to the disruption of the Spo7 interaction with Pah1. How lipin 1 interacts with the CTDNEP1–NEP1-R1 complex is unknown. Lack of an acidic tail in lipin 1 and a basic tail in NEP1-R1 indicates a different mechanism of interaction when compared with that of the Nem1–Spo7/Pah1 phosphatase cascade components. This highlights the fact that while the ultimate function of these phosphatase cascades is the same, namely to convert PA to DAG, the mechanisms and regulations involved are not the same. The acidic tail of Pah1 that is required for its interaction with the Nem1–Spo7 complex (35), the basic tail of Spo7 that is required for its interaction with Pah1 (this study), and the RP domain that controls the phosphorylation of Pah1 (79) are not conserved in the mammalian CTDNEP1–NEP1-R1/lipin 1 phosphatase cascade. Conversely, the M-LIP domain found within the large intrinsically disordered region of lipin 1 that is important for its dimerization and membrane association (80) is not found in Pah1. Whereas the Spo7 basic tail and Pah1 acidic tail, respectively, are not conserved in the mammalian NEP1-R1 and lipin 1 counterpart proteins, these regions are conserved in fungi, and in particular, some pathogenic ones that affect humans (*e.g.*, *Aspergillus nidulans*, *Candida albicans*, *Candida glabrata*) and plants (*Botryosphaeria dothidea*) (Fig. 11). Thus, the Spo7 basic and the Pah1 acidic tails are potential drug targets that will presumably not affect the function of the CTDNEP1–NEP1-R1/lipin 1 phosphatase cascade in higher eukaryotes.

## Experimental procedures

### Reagents

All chemicals were of reagent grade or of higher quality. Growth media were purchased from Difco Laboratories. Qiagen was the source of DNA and plasmid extraction kits. Clontech was the source of the carrier DNA for yeast transformation and DNA size markers. New England Biolabs supplied the enzyme reagents for DNA manipulation and the Q5 site-directed mutagenesis kit. Bio-Rad was the source of reagents used for Western blotting, Bradford protein assay reagent, and molecular mass protein standards. GE Healthcare was the source for polyvinylidene difluoride membrane, enhanced chemifluorescence substrate for Western blotting, and IgG-Sepharose beads. Silica gel 60 TLC plates, ampicillin, bovine serum albumin, 2-mercaptoethanol, PCR primers for mutagenesis, nucleotides, Triton X-100, protease inhibitors, rabbit anti–protein A antibody (product P3775, lot no.: 025K4777), and alkaline phosphatase–conjugated goat antimouse IgG antibodies (product number: A3562; lot no.: SLBG1482V) were purchased from Millipore–Sigma. Invitrogen was the source of mouse anti-Pgk1 antibodies (product number: 459250; lot no.: E1161). Thermo Fisher Scientific supplied alkaline phosphatase–conjugated goat anti-rabbit IgG antibody (product number: 31340; lot no.: NJ178812) and BODIPY 493/503. Radiochemicals and Scintillation counting supplies were obtained from PerkinElmer Life Sciences and National Diagnostics, respectively. BioSynthesis, Inc prepared the rabbit anti-Spo7 antibody directed against the amino acid sequence EDDLRRQAHEQK (residues 58-69) (43), rabbit anti-Pah1 antibody directed against the sequence TSIDKEFKKLSVSKAGA (residues 778-794) (24), and rabbit anti-Cho1 (phosphatidylserine synthase) antibody directed against the sequence MVESDEDFAPQEFPH (residues 1-15) (81). The IgG fraction of each antibody was isolated (82) from the serum and utilized in this study. Spo7 C-terminal WT (GGGGWAREGARRRKQAHELRPKSE, residues 240-259) and mutant (GGGGWAAEGAAAAAQAHELAPASE, residues 240-259) peptides and Pah1 C-terminal peptide (GGGGMDDKDLDSRVSDEFDDDEFDEDEFED, residues 838-862) were synthesized by ABclonal.

### Plasmids, strains, and DNA manipulations

The plasmids used in this study are listed in Table 1. Conventional procedures were utilized for the isolation of plasmid DNA and its manipulation (83, 84, 85). Transformation of *Escherichia coli* (83) and *S. cerevisiae* (86) with plasmid DNA was performed using standard protocols. Plasmid pGH443 (39), which is a derivative of pRS415 (87), directs the low-copy expression of *SPO7* from its own promoter in yeast. Plasmids YCplac111-*GAL1/10*-*NEM1-PtA* and pRS314-*GAL1/10*-*SPO7* were used for the galactose-induced overexpression of protein A-tagged Nem1 and Spo7, respectively. Derivatives of pGH443 and pRS314-*GAL1/10*-*SPO7* were constructed by Q5 site-directed mutagenesis with primers designed using the NEBaseChanger online program. All mutations were confirmed by DNA sequencing. Plasmid pGH450 contains the *SEC63*-*GFP* fusion inserted into pRS414 (87). Plasmid pGH452 bearing *PAH1*-PtA under the control of *GAL1* promoter was derived from a high-copy number *E. coli*/yeast shuttle vector, pYES2 (88).

The strains used in this study are listed in Table 1. *E. coli* strain DH5α was used for plasmid amplification and maintenance. All *S. cerevisiae* strains were derived from RS453 (89). GHY67 (39) is a *spo7*Δ*::URA3* mutant strain that was used for the plasmid-directed expression of WT and Spo7 mutant proteins. The *pah1*Δ*::natMX4* disruption cassette, which was generated by PCR amplification from pAG25 (EUROSCARF) as described previously for the *app1*Δ*::natMX4* cassette (90), was transformed into the *nem1*Δ *spo7*Δ mutant strain (SS1010) (30) to construct the *nem1*Δ *spo7*Δ *pah1*Δ mutant strain (GHY85) by one-step gene replacement (91). The nourseothricin (100 μg/ml)-resistant transformant cells were analyzed by PCR to confirm the gene replacement. The triple mutant was used for the overexpression of the protein A-tagged Nem1–Spo7 complex. The *pah1*Δ mutation prevents the growth inhibition caused by the overexpression of the protein phosphatase complex (31). The *pah1*Δ *nem1*Δ mutant (SS1132) (24) was used for pGH452-mediated overexpression of the phosphorylated Pah1 and its purification (88). The *nem1*Δ mutant cells lack the Nem1–Spo7 complex, ensuring the hyperphosphorylation of Pah1 (31, 88).

### Growth conditions

*E. coli* cells were cultured in Luria–Bertani broth (1% tryptone, 0.5% yeast extract, 1% NaCl, pH 7.0) containing 100 μg/ml ampicillin at 37 °C to select transformants carrying plasmids. Yeast cells were cultured using standard methods (83, 84); they were routinely grown at 30 °C in YPD (1% yeast extract, 2% peptone, and 2% dextrose) or SC media. Cells carrying plasmids were selected for or maintained by growth in SC medium without an appropriate amino acid. The media contained 2% dextrose as a carbon source, unless indicated otherwise. Bacterial and yeast growth in liquid medium was estimated spectrophotometrically by absorbance at 600 nm. For the temperature sensitivity assay, plasmid-carrying cells were serially diluted (10-fold) in SC-Leu media and spotted onto SC-Leu or YPD agar plates. Cell growth was assessed after 3 days of incubation at 30 and 37 °C. The growth patterns on each medium were similar; and the presented data were from the YPD plates. To induce the expression of protein A-tagged Nem1 and Spo7 (WT and mutant forms) with galactose, cells were first grown to the exponential phase in SC-Leu-Trp medium with 2% dextrose and then washed and resuspended in SC-Leu-Trp medium containing 2% galactose and 1% raffinose, followed by incubation for 14 h.

### Lipid labeling and analysis

*S. cerevisiae* cells were labeled to steady state with [2-^14^C]acetate (92); lipids were extracted from stationary phase cells by the Bligh and Dyer method (93) as described by Fakas *et al.* (94). The extracted lipids were separated by one-dimensional TLC on silica gel plates, utilizing the solvent system hexane/diethyl ether/glacial acetic acid (40:10:1, v/v) (95). After resolution, the lipids were visualized by phosphorimaging with the Storm 860 Molecular Imager (GE Healthcare) and analyzed by ImageQuant software (GE Healthcare). A standard curve of [2-^14^C]acetate was used for analysis. To confirm the identities of the radiolabeled TAG and total phospholipids, their migration on the silica gel was compared to authentic standards visualized by iodine vapor staining.

### Fluorescence microscopy

For the fluorescent staining of lipid droplets, *S. cerevisiae* cells were cultured in SC-Leu media at 30 °C to the stationary phase, incubated with 1 μg/ml BODIPY 493/503 for 30 min, washed with phosphate-buffered saline (pH 7.4), and resuspended in a small volume of the same buffer for imaging (42, 43). For the analysis of nuclear/ER membrane expansion, the yeast cells were transformed with *SEC63*-*GFP* plasmid, grown in SC-Leu-Trp to the logarithmic phase, and subjected to fluorescence microscopy analysis. The fluorescent signal from the lipid droplets and Sec63-GFP was examined under a Nikon Eclipse Ni-U microscope equipped with an enhanced GFP/FITC/Cy2/AlexaFluor 488 filter and recorded by a DS-Qi2 camera. Captured images were analyzed with the NIS-elements BR software. The number of lipid droplets per cell and the percentage of cells with aberrant nuclear/ER morphology (misshaped *versus* round nuclei) was determined by examination from ≥4 fields of view (≥200 cells).

### Preparation of cell extracts, membranes, and protein isolations

All procedures were conducted at 4 ^°^C. Yeast cultures were collected by centrifugation at 1500*g* for 5 min. The harvested cells were washed with water and resuspended in lysis buffer (50 mM Tris–HCl [pH 7.5], 10% glycerol, 10 mM 2-mercaptoethanol, 1 mM Na_2_EDTA, 0.5 mM phenylmethylsulfonyl fluoride, 1 mM benzamidine, 5 μg/ml aprotinin, 5 μg/ml leupeptin, and 5 μg/ml pepstatin). To disrupt the cells, glass beads (diameter of 0.5 mm) were added to the cell suspension, and the cell mixture was subjected to five repeats of 1 min burst of bead beating, followed by 2 min cooling using a BioSpec Products Mini-Beadbeater-16 (96). The cell lysates were centrifuged at 1500*g* for 10 min to separate unbroken cells and cell debris from cell extracts (supernatant). The cell extract was centrifuged at 100,000*g* for 1 h to obtain the membrane fraction (pellet). The membrane fraction, which was used for the Nem1–Spo7 protein phosphatase assay, was resuspended in 50 mM Tris–HCl buffer (pH 7.5) containing 10 mM MgCl_2_, 10 mM 2-mercaptoethanol, 10% glycerol, and protease inhibitors.

Protein A-tagged Nem1–Spo7 complex was isolated from 250 ml cultures of the *nem1*Δ *spo7*Δ *pah1*Δ triple mutant (GHY85) expressing plasmids YCplac111-*GAL1/10*-*NEM1*-PtA and pRS314-*GAL 1/10-SPO7* (WT or C-terminal region mutant forms) using IgG-Sepharose by the method of Siniossoglou *et al.* (97) with minor modifications (33). The amount of the complex proteins isolated was not sufficient for detection by protein staining on an SDS-polyacrylamide gel. Instead, isolated Nem1-PtA and Spo7 proteins were detected by immunoblotting with anti-protein A and anti-Spo7 antibodies, respectively. Phosphorylated Pah1 was isolated from the *pah1*Δ *nem1*Δ double mutant (SS1132) expressing plasmid pGH452 using a combination of IgG-Sepharose chromatography, anion exchange chromatography, and size-exclusion chromatography (88). The purity of the Pah1 preparation was the same as that shown previously (88). The protein preparations were stored at −80 °C.

### SDS-PAGE and immunoblot analysis

Conventional protocols were employed for SDS-PAGE (98) and immunoblotting using a polyvinylidene difluoride membrane (99, 100). The samples for immunoblotting were normalized based on total protein loading. Protein transfer from polyacrylamide gels to polyvinylidene difluoride membranes was monitored by staining with Ponceau S. The blots were probed with rabbit anti-protein A (1 μg/ml), rabbit anti-Spo7 (1 μg/ml), rabbit anti-Pah1 (2 μg/ml), rabbit anti-Cho1 (0.25 μg/ml), or mouse anti-Pgk1 (2 μg/ml) antibody. Alkaline phosphatase–conjugated goat anti-rabbit IgG antibody and goat antimouse IgG antibody were used at a dilution of 1:5000. Immune complexes were detected using an enhanced chemifluorescence substrate for alkaline phosphatase. Fluorescence signals from immunoblots were acquired with a Storm 860 Molecular Imager, and the signal intensities were analyzed by ImageQuant TL software. A standard curve was used to ensure that the immunoblot signals were in the linear range of detection.

### Nem1–Spo7 protein phosphatase assay

The protein phosphatase activity of the Nem1–Spo7 complex in the membrane fraction, obtained from the GHY85 cells expressing protein A-tagged Nem1 and Spo7 (WT or C-terminal region mutant forms), was evaluated using electrophoretic mobility of Pah1 on 6% polyacrylamide gels (42, 43). The reaction mixture consisted of 100 mM sodium acetate (pH 5.0), 10 mM MgCl_2_, 1 mM DTT, 20 μg membranes, and 2.5 ng Pah1 in a total volume of 20 μl.

### Glutaraldehyde crosslinking of peptides

Peptides were crosslinked with glutaraldehyde as described by Liao *et al.* (59). Spo7 (WT or mutant) peptide (20 μg) was incubated with Pah1 peptide (20 μg) in 5 μl of 100 mM potassium phosphate buffer (pH 6.5) containing 5 mM glutaraldehyde for 1 h at room temperature. Following the crosslinking, the samples were mixed with 5 μl of 150 mM Tris–HCl (pH 7.0) buffer containing 12% SDS, 6% 2-mercaptoethanol, 30% glycerol, and 0.05% Coomassie blue G-250 and incubated for 1 h at 37 ^°^C. The samples were then analyzed by Tris–tricine-PAGE (16.5% polyacrylamide gel) as described previously (101). The polyacrylamide gel was stained with SYPRO Ruby; cross-linked peptides were visualized by fluorescence imaging with the Storm 865 Molecular Imager.

### Protein determination

Protein was estimated by the method of Bradford (102) using bovine serum albumin as the standard.

### Data analysis

Statistical analyses were performed with Microsoft Excel software. *p* Values <0.05 were taken as a significant difference.

## Data availability

All data are contained within the article.

## Conflict of interest

The authors declare that they have no conflicts of interest with the contents of this article.
